# Supplementary material for: Supporting COVID-19 policy-making with a predictive epidemiological multi-model warning system
Source: Commun Med (Lond). 2022 Dec 8;2:157. doi: 10.1038/s43856-022-00219-z (PMC9729177; doi:10.1038/s43856-022-00219-z)
Supplement: Supplementary file 2 — Supplementary Information [file 43856_2022_219_MOESM2_ESM.pdf]

Supplementary Material

SUPPLEMENTARY NOTE 1: DETAILS ON THE EXTENDED SIR-X MODEL

The SIR-X model [1] was originally devised to model the transition from exponential to sub-exponential growth due to the implementation of non-pharmaceutical interventions (NPIs). To also account for the taking back of NPIs, a couple of modifications are therefore needed to the original model. In the following we give a description of how we extended the baseline SIR-X model in order to implement such processes. We also discuss additional extensions to the model, such as introducing age-structured populations and multiple calibration periods. Note that this document only discusses aspects of the model implementation not described or treated different than in the original model description. The model has been individually calibrated to each of the nine federal states of Austria.

A. Compartments for quarantined infected and susceptibles

The baseline model includes two different types of NPI. First, there are NPIs that act on the susceptible population (social distancing, home office, etc.). Second, there are NPIs that act on the infected population, in particular an accelerated detection of cases (e.g., testing and contact tracing). Clearly, easing of NPIs affects primarily the first type of NPIs, while it might be reasonable to expect that NPIs targeting the infected population might even increase in effectiveness.

The baseline SIR-X model is of the following form,

$$\partial_t S = -\alpha S I - \kappa_0 S \tag{1}$$

$$\partial_t I = \alpha S I - \beta I - \kappa_0 I - \kappa I \tag{2}$$

$$\partial_t R = \beta I + \kappa_0 S \tag{3}$$

$$\partial_t X = (\kappa + \kappa_0) I \quad . \tag{4}$$

We now introduce the following extensions, namely (i) having two compartments of contact-reduced individuals (susceptibles, X^S, and infected, i.e. isolated, X^I) (ii) introducing an easing of NPIs affecting susceptibles encapsulated in the rate $\kappa_1 \geq 0$, (iii) waning immunity (parameter γ) as well as (iv) an age structure by labelling the age strata with index a. The

extended SIR-X model is then of the following form,

$$\partial_t S_a = -\alpha c_{aa'} S_a I_{a'} - \kappa_0 S_a + \kappa_1 X_a^S + \gamma R_a \tag{5}$$

$$\partial_t I_a = \alpha c_{aa'} S_a I_{a'} - \beta I_a - \kappa_0 I_a - \kappa I_a \tag{6}$$

$$\partial_t R_a = \beta I_a - \gamma R \tag{7}$$

$$\partial_t X_a^I = \kappa I_a \tag{8}$$

$$\partial_t X_a^S = \kappa_0 S_a - \kappa_1 X_a^S \quad . \tag{9}$$

The compartment X^I is the *cumulative number of confirmed cases*; it will be used to calibrate the model. It is imperative to note that the model was explicitly designed to make statements concerning X^I. The compartment X^S is now an explicit model representation of locked down or socially distanced susceptibles. The parameter κ_0 gives the inflow to this compartment from the susceptibles (strength of corresponding NPIs), κ_1 gives the outflow (how fast people increase their levels of social contacts back to normal).

The matrix $c_{aa'}$ describes social mixing across age groups and gives the probability that a contact (that is relevant for disease transmission) of a potential infectee of age a will be with an infector of age a', i.e. we have $\sum_{a'} c_{aa'} = 1$. In the model we worked with four age groups covering the population between 0y and 99y in intervals of 25y.

B. Calibration

Calibration of the model parameters requires almost real-time information on social behaviour. We therefore used aggregate measures obtained from telecommunications data from which we continuously derived indicators for the level of mobility in specific regions and population groups; see [2, 3] for a technical description on how the telecommunication data we processed. In particular, we monitored the median radius of gyration as a mobility indicator and published results for these indicators periodically on our institute's website[4]. Furthermore, information on social mixing by age, the matrix $c_{aa'}$, has been obtained from aggregated call detail records in which the entry $c_{aa'}$ is the probability that when someone from age group a receives a call, the caller is a person from age group a'.

We acknowledge the limitation that phone calls are only a crude proxy for the measurement of face-to-face contacts but work with the assumption that two persons are much more

likely to have close physical contact with each other if they regularly call each other compared to two individuals that never exchange phone calls. Further, this procedure allows us to measure social mixing near real-time (typically, the mobility indicators and the age matrix $c_{aa'}$ were available with a lag of two to three days), as particularly in the early phases of the pandemic it was not clear how representative social mixing data obtained via surveys from before the pandemic was for the current mixing behaviour in the society varying NPI regimes.

Calibration for all model parameters except α and β takes place in different time windows that roughly represent the different phases of the epidemic in Austria. The first phase lasts from $t = 0$ to end of March and encompasses the "first wave". With beginning of April, Austria moved into a containment phase characterized by less than hundred new confirmed cases per day. The second calibration phase ends mid-June where the daily cases started to increase again with most days showing more than hundred new cases. The third calibration phase lasts until the end August, when infection numbers started to increase again, after which the fourth calibration phase commenced. The exact points upon which new calibration time windows should be started were corroborated with the time-series of the radius of gyration. Whenever this indicator showed a clear turning point signalling a substantial change in how people react relative to the current NPI regime, we started a new calibration window. In almost all cases this tracked closely with implementations and easings of lockdowns, as previously described [2, 3].

Calibration is finally performed via the timeseries of the cumulative number of confirmed cases $X^I = \sum_a X_a^I$ in each federal state of Austria and each time window. The basic calibration procedure follows [1] alongside with the values for α and β, i.e. we solve the model for solutions to the model parameters δ, κ, κ_0 and κ_1, as well as the initial condition $I(t = 0)$ via a trust region reflective algorithm (MatLab's lsqnonlin function), where we seek to minimize the mean squared error between the model and observed confirmed cases.

SUPPLEMENTARY NOTE 2: DETAILS ON THE EPIDEMIOLOGICAL STATE SPACE MODEL

The key idea of the model is to identify the trend in the Austrian Coronavirus case numbers by isolating exogenous shocks, exogenous covariates and weekday effects from the reported case numbers.

The model is implemented as a state-space model with exogenous data that takes logarithmized case numbers (y_t) as observations and links them to a state vector of length 8, which represents true level (x_t^1), trend (x_t^2), and weekday effects x_t^{3-8} of y_t. Additionally, exogenous variables that may affect the observations or the states are included.

The value of interest is (x_t^2) the *trend* state in the state space model, i.e. the parameter that describes the growth of the (true) case numbers and quantifies the local transmission activity. The current trend and the effects from the covariates determine the forecast. This annex gives details on how the model is specified and how external factors are identified and represented in the model.

C. Specification and fitting of the model

The model is specified and fitted using the R-package MARSS [5, 6]. This package allows fitting multivariate auto-regressive state space models with exogenous variables.

The basic structure of a MARSS is given by the following set of equations.

$$x_t = Bx_{t-1} + Cc_t + w_t, \text{ where } w_t \sim MVN(0, Q) \text{ and } x_t = x_t^1 \dots x_t^8 \tag{10}$$

$$y_t = Zx_t + a_t + v_t, \text{ where } v_t \sim MVN(0, R) \tag{11}$$

$$x_0 \sim MVN(\pi, \Lambda) \tag{12}$$

where y_t are observations; x_t are states; c_t and a_t are exogenous inputs; B, C, Z are parameter matrices; π and Λ represent starting values and variance of the states that are estimated in the model; and Q and R are diagonal matrices of variances that are estimated in the model.

In the model, the observations y_t are the logarithmic newly reported cases. The state vector is of length 8. The first state (x_t^1) represents the true "level" of daily new infections, i.e. after controlling for weekday effects x_t^{3-8}, idiosyncratic measurement error v_t and systematic

measurement error a_t. The second state x_t^2 represents the trend in daily infections and is the primary variable of interest. States 3 to 8 represent weekday effects. Matrix B links x_t to x_{t-1} and given by a combination of a random walk (RW) of order 2 for x^{1-2} as well as the sequence of weekday effects x^{3-8} and is given by:

$$B = \begin{pmatrix} 1 & 1 & 0 & 0 & 0 & 0 & 0 & 0 \\ 0 & 1 & 0 & 0 & 0 & 0 & 0 & 0 \\ 0 & 0 & -1 & -1 & -1 & -1 & -1 & -1 \\ 0 & 0 & 1 & 0 & 0 & 0 & 0 & 0 \\ 0 & 0 & 0 & 1 & 0 & 0 & 0 & 0 \\ 0 & 0 & 0 & 0 & 1 & 0 & 0 & 0 \\ 0 & 0 & 0 & 0 & 0 & 1 & 0 & 0 \\ 0 & 0 & 0 & 0 & 0 & 0 & 1 & 0 \end{pmatrix} \tag{13}$$

Matrix Z links states x_t to observations y_t and is thus given by:

$$Z = \begin{pmatrix} 1 & 0 & 1 & 0 & 0 & 0 & 0 & 0 \end{pmatrix} \tag{14}$$

In our model, the data is transformed to represent changes in the relevant state or observation vector. The matrix C links the column of the data matrix to the relevant state vector, which is the "trend" state for changes in immunization rates, NPIs, seasonality and regional convergence and the *level* state for changes in the share of imported cases.

Since each regional model only has one observation vector, and there is only one exogenous variable affecting the link between observations and the level state (i.e. the detection rate), we encode changes in the detection rate in the model parameter a_t.

The R-package MARSS is used to fit the model using the Broyden-Fletcher-Goldberg-Shannon (BFGS) Quasi-Newton algorithm.

D. Exogenous shocks

We define exogenous shocks as events which affect the reported case numbers but do not represent a corresponding change in transmission activity. For example, the introduction of a mass screening program is expected to increase detection rates and lead to higher reported case numbers but this increase in the number of identified cases does not signal immediate epidemiological changes. Likewise, import of a large number of cases that have acquired

the infection abroad following periods of increased travel activity increases reported case numbers.

In both examples, the trend in new cases is inflated above what can be attributed to reproduction of the Virus by local transmission; and forecasts based on the trend in new cases are biased upwards. In correction of that phenomenon, we distinguish three cases:

1. Temporary or permanent changes in the detection rate are modelled as temporary or constant effects on the relationship between the *level* state and the observations. For example, an increase in the detection rate would result in an increase in a_t.

2. An increase in active cases imported from abroad increases the observed daily case numbers but not does not necessarily change the transmission rates. A temporary change in the rate of imported cases is modelled by an exogenous variable that adds a certain number of cases to the *level* state.

3. Artificial shocks may also be created due to poor reporting. To anticipate reporting delays we apply the following nowcasting approach: for each federal state, weekday and lag (1 to 4 days) a nowcasting factor is calculated by taking the group-specific mean of the ratio of the revised (i.e., after reported cases have been back-filled) and the initial daily case number of the previous four weeks. The adjusted time series is used for the model.

Isolation of exogenous shocks works best if case numbers are low and information from contact tracing is available in a timely manner. We extract this information from cluster analyses of the Austrian Agency for Health and Food Safety [7].

E. Weekday effects

We model weekend effects as a set of 6 states that affect the "level" state. Weekend effects may vary over time in a first order random-walk (RW1) model, the variance of which is identical for all weekend effects in a region and estimated in the model. The specification of the weekend effects in the state space model follows the dynamic linear model *dlmModSeas* in the *dlm* R-package [8, 9]; with the exception that weekend effects' state noise is not set to zero.

F. Changes in NPI

Known changes in NPI explain both regional and temporal differences in transmission activity. We model the effect of NPI on the *trend* state via an exogenous variable that increments or decrements the *trend* state of a region by a specified amount. The extent of that effect is estimated based on literature regarding the effect of NPIs (e.g. [10]). This effect is spread over a period of 10 days.

G. Changes in immunization

Both vaccination and recovery reduce the share of population susceptible for infection with SARS-CoV-2, albeit to a varying degree. We model the effect of immunization by calculating the percentage of people who are effectively protected from infection in a region. This figure is generated from the number of vaccinated multiplied by vaccine effectiveness plus the number of recovered multiplied by recovery effectiveness. Effectiveness of vaccination and recovery with regard to infection probability is extracted from ratios of grouped incidence rates ("screening method"). These effectiveness figures are updated for every forecast and thus reflect both waning vaccine effectiveness and loss of natural immunity after recovery as evident in the Austrian incidence data. The share of people effectively vaccinated can be easily determined for the time frame of the forecast, since immunization is considered to be in effect starting two weeks after the second dose.

We assume that a 1% increase in the share of people effectively protected cases approximately a 1% decrease in the effective reproduction number. We compute a corresponding effect on the *trend* state and code it an external variable.

H. Seasonal effects

Our model includes seasonality by including average UV index over time as an exogenous factor, which is lowest in beginning of January, highest in beginning of July and has a sinusoidal shape. [citation needed] Following the literature, we assume an effect of $\pm 15\%$ on the effective reproduction number due to seasonality. We therefore construct a covariate that adjusts the *trend* state by the daily difference ($\Delta_{seas,t}$) for each day of the year (yd) as follows:

$$\Delta_{seas,t} = 0.15\left(\cos(2\pi yd_t/365) - \cos(2\pi yd_{t-1}/365)\right) \tag{15}$$

The effect of this seasonal component has its largest effect on the *trend* state in mid-October and mid-March but can be considered rather small in a given forecast that only spans several days.

I. Regional convergence

Regional interdependence is modelled by assuming a degree of regional convergence in the model. Due to computational efficiency, we fit the model separately for all regions as well as for the whole country in a first step. We compute expected differences in trends due to regional differences (NPIs, share of immunized population, ...) and assume that regional trends converge to the national trend (adjusted by the aforementioned expected differences). Convergence is implemented by taking the average of the regional and national *trend* state, controlling for region-specific NPI.

J. Strengths and limitations

The Epidemiological State Space Model tries to identify and perpetuate the current trend of local transmission activity by isolating the effects of factors such as seasonality, NPI, weekday effects and immunization. The model however does not determine the individual effects of these contributing factors.

The advantage of the model lies in its comparable small set of assumptions in the model specification and parsimonious nature of time series smoothing and trend extrapolation. Drawing from the rapidly growing literature on effect sizes of NPI and seasonality, researchers do not need to make their own assumptions on these factors. However, the mapping of current and planned NPI in Austria to effect sizes drawn from literature is challenging and relies on subjective assessments based on qualitative data such as adherence of the population to NPI. Thus, such assessments are crucial for the prediction of turning points based on NPIs.

SUPPLEMENTARY REFERENCES

[1] B. F. Maier and D. Brockmann, Effective containment explains subexponential growth in recent confirmed covid-19 cases in china, Science **368**, 742 (2020).

[2] G. Heiler, T. Reisch, J. Hurt, M. Forghani, A. Omani, A. Hanbury, and F. Karimipour, Country-wide mobility changes observed using mobile phone data during covid-19 pandemic, arXiv preprint arXiv:2008.10064 (2020).

[3] T. Reisch, G. Heiler, J. Hurt, P. Klimek, A. Hanbury, and S. Thurner, Behavioral gender differences are reinforced during the covid-19 crisis, Scientific reports **11**, 1 (2021).

[4] `https://www.csh.ac.at/wp-content/uploads/2021/01/2021-01-25-CSH-Policy-Brief-Bewegungsradi` `pdf`, accessed April 1, 2022.

[5] E. Holmes, E. Ward, M. Scheuerell, and K. Wills, *MARSS: Multivariate Autoregressive State-Space Modeling* (2020), r package version 3.11.3.

[6] E. E. Holmes, E. J. Ward, and K. Wills, Marss: Multivariate autoregressive state-space models for analyzing time-series data, The R Journal **4**, 30 (2012).

[7] Austrian Agency for Health and Food Safety, Epidemiologische Abklärung Covid 19. Fallabklärung, `https://www.ages.at/themen/krankheitserreger/coronavirus/epidemiologische-abklaerung-covid-19/`, accessed: 2021-09-20.

[8] G. Petris, S. Petrone, and P. Campagnoli, *Dynamic Linear Models with R*, useR! (Springer-Verlag, New York, 2009).

[9] G. Petris, An R package for dynamic linear models, Journal of Statistical Software **36**, 1 (2010).

[10] N. Haug, L. Geyrhofer, A. Londei, E. Dervic, A. Desvars-Larrive, V. Loreto, B. Pinior, S. Thurner, and P. Klimek, Ranking the effectiveness of worldwide COVID-19 government interventions, Nature Human Behaviour **4**, 1303 (2020).

[11] D. W. Scott, *Multivariate density estimation: theory, practice, and visualization* (John Wiley & Sons, 2015).

[12] A. F. M. of Health, Diagnosen- und Leistungsdokumentation des Bundesministeriums für Soziales, Gesundheit, Pflege und Konsumentenschutz.

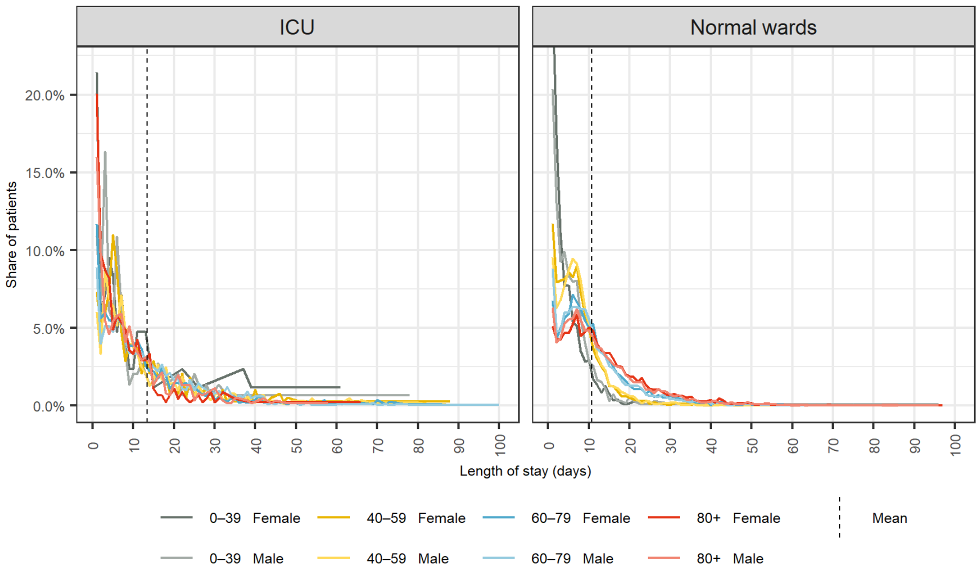

Supplementary Figure 1. Distribution of length of stay of COVID-19 patients admitted to ICU and normal wards based on Austrian hospital billing data [12] (Admissions from January to May 2021).

**Sex**	**Age group**	normal ward	ICU
Male	0–39	0.92	0.10
Male	40–59	5.17	1.03
Male	60–79	20.62	5.00
Male	80+	42.06	4.75
Female	0–39	0.83	0.06
Female	40–59	2.90	0.37
Female	60–79	15.42	2.54
Female	80+	29.46	1.89

Supplementary Table I. COVID-19 hospitalisation rate for ICU and normal wards as percentage of detected cases, by age and sex based on Austrian hospital billing data [12] (Admissions from January to May 2021).

**Parameter**	**Value**
Share of normal ward patients with positive test and admissions on the same day	80%
Share of ICU patients with positive test and admissions on the same day	30%
Maximum Time from positive test to hospitalisation	4 days
Maximum Time from positive test to ICU admission	7 days
Share of ICU patients with subsequent normal ward stay	75%
Length of normal ward stay after ICU stay	7 days

Supplementary Table II. Model parameters for the hospital bed usage model
