# Supplementary material for: Supporting COVID-19 policy-making with a predictive epidemiological multi-model warning system
Source: Commun Med (Lond). 2022 Dec 8;2:157. doi: 10.1038/s43856-022-00219-z (PMC9729177; doi:10.1038/s43856-022-00219-z)
Supplement: Supplementary file 3 — Description of Additional Supplementary Files [file 43856_2022_219_MOESM3_ESM.docx]

**Description of Additional Supplementary Files**

**File name:** Supplementary Data 1
**Description:** Data for Figure 1

**File name:** Supplementary Data 2
**Description:** Data for Figure 2

**File name:** Supplementary Data 3
**Description:** Data for Figure 3

**File name:** Supplementary Data 4
**Description:** Data for Figure 4
